# 4-Amino-12-methyl­sulfon­yloxy-[2.2]para­cyclo­phane

**DOI:** 10.1107/S1600536813032595

**Published:** 2013-12-07

**Authors:** Xiangchao Meng, Wenzeng Duan, Yinfeng Han

**Affiliations:** a School of Chemistry and Chemical Engineering, Shandong University, Jinan 250100, People’s Republic of China; b School of Chemistry and Chemical Engineering, Taian University, Taian 271021, People’s Republic of China; c School of Chemistry and Chemical Engineering, Liaocheng University, Liaocheng 252000, People’s Republic of China

## Abstract

The title compound, C_17_H_19_NO_3_S, was synthesized from 4-benzhydryl­idene­amino-12-hy­droxy-[2.2]para­cyclo­phane and methane­sulfonyl chloride. In the mol­ecule, the distance between the centroids of two aromatic rings is 2.960 (5) Å. In the crystal, weak N—H⋯O and C—H⋯O hydrogen bonds link the mol­ecules into layers parallel to the *ac* plane.

## Related literature   

For background to [2.2]para­cyclo­phane, see: Cram *et al.* (1959 ▶); Liebman & Greenberg (1976 ▶); Dyson *et al.* (1998 ▶). For its synthesis and applications in catalysis, see: Hou *et al.* (2000 ▶); Duan *et al.* (2008 ▶, 2012 ▶). For a related structure, see: Ma *et al.* (2012 ▶).
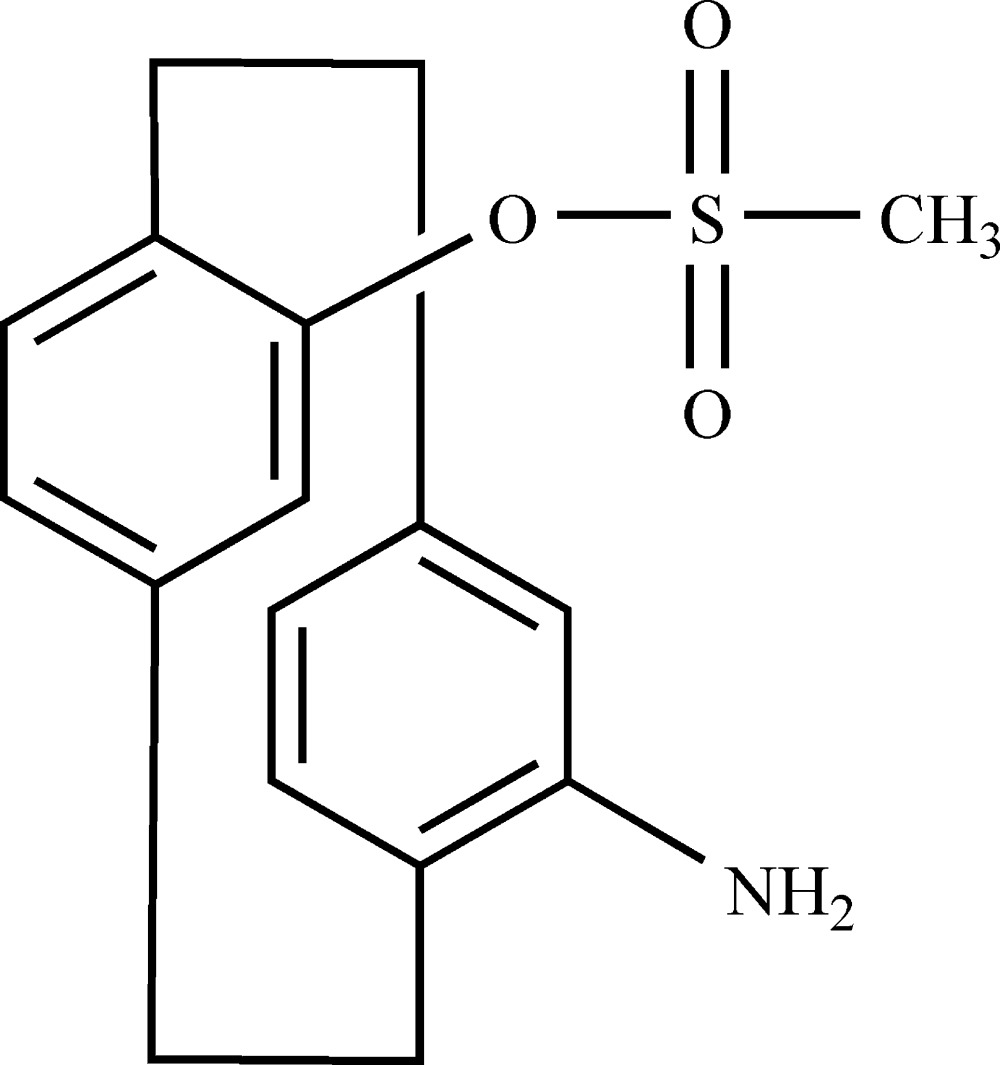


## Experimental   

### 

#### Crystal data  

C_17_H_19_NO_3_S
*M* *_r_* = 317.39
Orthorhombic, 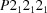

*a* = 8.017 (7) Å
*b* = 11.734 (9) Å
*c* = 16.131 (13) Å
*V* = 1517 (2) Å^3^
*Z* = 4
Mo *K*α radiation
μ = 0.23 mm^−1^
*T* = 273 K
0.13 × 0.12 × 0.10 mm

#### Data collection  

Bruker SMART CCD diffractometer
Absorption correction: multi-scan (*SADABS*; Bruker, 2007 ▶) *T*_min_ = 0.971, *T*_max_ = 0.978
7769 measured reflections
2676 independent reflections
2266 reflections with *I* > 2σ(*I*)
*R*_int_ = 0.037

#### Refinement  

*R*[*F* ^2^ > 2σ(*F* ^2^)] = 0.038
*wR*(*F* ^2^) = 0.084
*S* = 1.04
2676 reflections
200 parameters
H-atom parameters constrained
Δρ_max_ = 0.15 e Å^−3^
Δρ_min_ = −0.23 e Å^−3^
Absolute structure: Flack (1983 ▶), 1122 Friedel pairs
Absolute structure parameter: 0.02 (9)

### 

Data collection: *SMART* (Bruker, 2007 ▶); cell refinement: *SAINT* (Bruker, 2007 ▶); data reduction: *SAINT*; program(s) used to solve structure: *SHELXS97* (Sheldrick, 2008 ▶); program(s) used to refine structure: *SHELXL97* (Sheldrick, 2008 ▶); molecular graphics: *SHELXTL* (Sheldrick, 2008 ▶); software used to prepare material for publication: *SHELXTL*.

## Supplementary Material

Crystal structure: contains datablock(s) I, global. DOI: 10.1107/S1600536813032595/cv5436sup1.cif

Structure factors: contains datablock(s) I. DOI: 10.1107/S1600536813032595/cv5436Isup2.hkl

Additional supporting information:  crystallographic information; 3D view; checkCIF report

## Figures and Tables

**Table 1:** Hydrogen-bond geometry (Å, °)

*D*—H⋯*A*	*D*—H	H⋯*A*	*D*⋯*A*	*D*—H⋯*A*
N1—H1*B*⋯O2^i^	0.86	2.43	3.262 (3)	163
C10—H10*B*⋯O1^ii^	0.97	2.52	3.390 (4)	149

Symmetry codes: (i) 
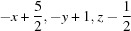
; (ii) 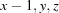.

**Fig. 1.**
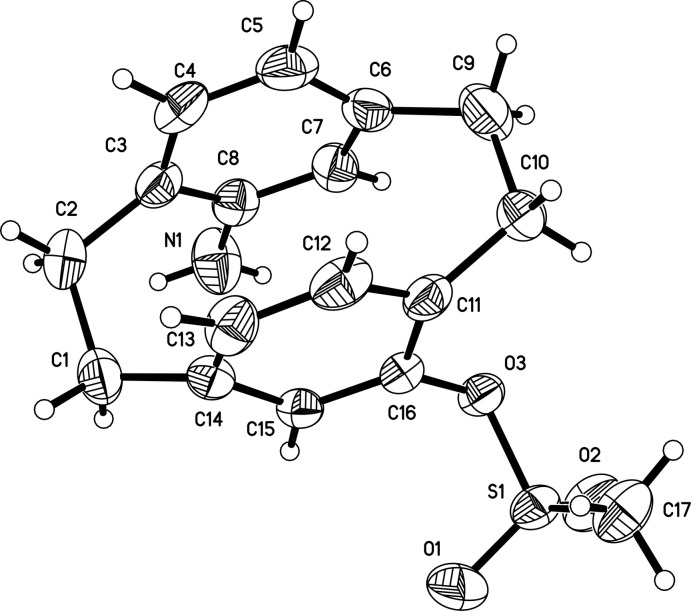
The molecular structure of the title compound showing the atom-numbering scheme and 50% probability displacement ellipsoids.

## supplementary crystallographic information

### 1. Comment

Since the first synthesis of [2.2]paracyclophane (Cram, 1959), its structure atrracted considerable interest (Liebman *et al.*, 1976; Dyson *et al.*, 1998). [2.2]Paracyclophane needs only one substituent to become planar chiral, so, there has been notable progress with regard to the synthesis of new derivatives and their applications in asymmetric catalysis(Hou *et al.*, 2000; Duan *et al.*, 2008).

In the title compound (Fig. 1), all bond lengths and angles are normal and in agreement with those observed in the related structure (Ma *et al.*, 2012). In the molecule, the distance between the centroids of two aromatic rings is 2.960 (5) Å. The crystal packing exhibits weak intermolecular N—H···O and C—H···O hydrogen bonds (Table 1), which link the molecules into layers parallel to the *ac* plane.

### 2. Experimental

The title compound was prepared by the method reported by Duan *et al.* (2012). The crystals were obtained by recrystallization from hexane and ethyl acetate.

### 3. Refinement

All the H atoms were located in difference maps, but placed in idealized positions (N—H 0.86 Å, C—H 0.93–0.97 Å), and refined as riding, with with *U*_iso_(H) = 1.2–1.5 *U*_eq_ of the parent atom.

### Crystal data

**Table:**

C_17_H_19_NO_3_S	*F*(000) = 672
*M**_r_* = 317.39	*D*_x_ = 1.389 Mg m^−^^3^
Orthorhombic, *P*2_1_2_1_2_1_	Mo *K*α radiation, λ = 0.71073 Å
Hall symbol: P 2ac 2ab	Cell parameters from 2210 reflections
*a* = 8.017 (7) Å	θ = 2.8–23.1°
*b* = 11.734 (9) Å	µ = 0.23 mm^−^^1^
*c* = 16.131 (13) Å	*T* = 273 K
*V* = 1517 (2) Å^3^	Block, colourless
*Z* = 4	0.13 × 0.12 × 0.10 mm

### Data collection

**Table:**

Bruker SMART CCD diffractometer	2676 independent reflections
Radiation source: fine-focus sealed tube	2266 reflections with *I* > 2σ(*I*)
Graphite monochromator	*R*_int_ = 0.037
phi and ω scans	θ_max_ = 25.0°, θ_min_ = 2.2°
Absorption correction: multi-scan (*SADABS*; Bruker, 2007)	*h* = −9→9
*T*_min_ = 0.971, *T*_max_ = 0.978	*k* = −13→13
7769 measured reflections	*l* = −11→19

### Refinement

**Table:**

Refinement on *F*^2^	Secondary atom site location: difference Fourier map
Least-squares matrix: full	Hydrogen site location: inferred from neighbouring sites
*R*[*F*^2^ > 2σ(*F*^2^)] = 0.038	H-atom parameters constrained
*wR*(*F*^2^) = 0.084	*w* = 1/[σ^2^(*F*_o_^2^) + (0.0436*P*)^2^ + 0.0218*P*] where *P* = (*F*_o_^2^ + 2*F*_c_^2^)/3
*S* = 1.04	(Δ/σ)_max_ < 0.001
2676 reflections	Δρ_max_ = 0.15 e Å^−^^3^
200 parameters	Δρ_min_ = −0.23 e Å^−^^3^
0 restraints	Absolute structure: Flack (1983), 1122 Friedel pairs
Primary atom site location: structure-invariant direct methods	Absolute structure parameter: 0.02 (9)

### Special details

**Table:**

Geometry. All e.s.d.'s (except the e.s.d. in the dihedral angle between two l.s. planes) are estimated using the full covariance matrix. The cell e.s.d.'s are taken into account individually in the estimation of e.s.d.'s in distances, angles and torsion angles; correlations between e.s.d.'s in cell parameters are only used when they are defined by crystal symmetry. An approximate (isotropic) treatment of cell e.s.d.'s is used for estimating e.s.d.'s involving l.s. planes.
Refinement. Refinement of *F*^2^ against ALL reflections. The weighted *R*-factor *wR* and goodness of fit *S* are based on *F*^2^, conventional *R*-factors *R* are based on *F*, with *F* set to zero for negative *F*^2^. The threshold expression of *F*^2^ > σ(*F*^2^) is used only for calculating *R*-factors(gt) *etc*. and is not relevant to the choice of reflections for refinement. *R*-factors based on *F*^2^ are statistically about twice as large as those based on *F*, and *R*- factors based on ALL data will be even larger.

### Fractional atomic coordinates and isotropic or equivalent isotropic displacement parameters (Å^2^)

**Table:**

	*x*	*y*	*z*	*U*_iso_*/*U*_eq_	
C1	1.0276 (4)	0.2690 (2)	0.10319 (15)	0.0464 (7)	
H1C	1.0358	0.1905	0.0849	0.056*	
H1D	1.1386	0.3019	0.1019	0.056*	
C2	0.9100 (3)	0.3373 (2)	0.04181 (16)	0.0484 (7)	
H2A	0.9781	0.3785	0.0022	0.058*	
H2B	0.8411	0.2839	0.0113	0.058*	
C3	0.7987 (3)	0.4208 (2)	0.08747 (14)	0.0386 (6)	
C4	0.6334 (4)	0.3938 (2)	0.10371 (16)	0.0478 (7)	
H4	0.5785	0.3439	0.0682	0.057*	
C5	0.5469 (3)	0.4382 (2)	0.17062 (18)	0.0492 (7)	
H5	0.4357	0.4188	0.1794	0.059*	
C6	0.6281 (3)	0.5120 (2)	0.22456 (16)	0.0412 (6)	
C7	0.7798 (3)	0.5569 (2)	0.19893 (15)	0.0402 (6)	
H7	0.8257	0.6175	0.2283	0.048*	
C8	0.8654 (3)	0.5139 (2)	0.13052 (15)	0.0376 (6)	
C9	0.5778 (4)	0.5230 (3)	0.31434 (17)	0.0551 (8)	
H9A	0.4571	0.5206	0.3180	0.066*	
H9B	0.6140	0.5967	0.3348	0.066*	
C10	0.6529 (3)	0.4269 (2)	0.37183 (16)	0.0491 (7)	
H10A	0.7088	0.4625	0.4185	0.059*	
H10B	0.5624	0.3808	0.3935	0.059*	
C11	0.7741 (3)	0.3510 (2)	0.32766 (15)	0.0359 (6)	
C12	0.7184 (3)	0.2616 (2)	0.27724 (15)	0.0425 (7)	
H12	0.6159	0.2280	0.2886	0.051*	
C13	0.8110 (3)	0.2222 (2)	0.21135 (16)	0.0432 (7)	
H13	0.7716	0.1612	0.1802	0.052*	
C14	0.9620 (3)	0.27203 (19)	0.19075 (14)	0.0358 (6)	
C15	1.0336 (3)	0.34300 (19)	0.24992 (15)	0.0336 (6)	
H15	1.1437	0.3666	0.2440	0.040*	
C16	0.9421 (3)	0.37825 (19)	0.31712 (14)	0.0304 (6)	
C17	1.0148 (4)	0.3455 (2)	0.51017 (17)	0.0548 (8)	
H17A	0.9649	0.2812	0.4830	0.082*	
H17B	0.9287	0.3953	0.5303	0.082*	
H17C	1.0816	0.3196	0.5558	0.082*	
N1	1.0226 (3)	0.5546 (2)	0.11279 (15)	0.0610 (7)	
H1A	1.0668	0.6060	0.1438	0.073*	
H1B	1.0764	0.5286	0.0707	0.073*	
O1	1.2545 (2)	0.34091 (17)	0.40407 (12)	0.0602 (6)	
O2	1.2004 (2)	0.52194 (15)	0.47560 (11)	0.0553 (5)	
O3	1.0125 (2)	0.46195 (13)	0.37118 (10)	0.0355 (4)	
S1	1.14007 (8)	0.41905 (5)	0.43999 (4)	0.03807 (18)	

### Atomic displacement parameters (Å^2^)

**Table:**

	*U*^11^	*U*^22^	*U*^33^	*U*^12^	*U*^13^	*U*^23^
C1	0.0554 (18)	0.0443 (15)	0.0395 (15)	0.0054 (14)	0.0040 (13)	−0.0094 (12)
C2	0.059 (2)	0.0540 (16)	0.0325 (15)	−0.0026 (14)	0.0012 (12)	−0.0085 (12)
C3	0.0400 (15)	0.0459 (14)	0.0298 (13)	−0.0008 (13)	−0.0051 (10)	0.0041 (11)
C4	0.0484 (17)	0.0558 (17)	0.0393 (15)	−0.0035 (15)	−0.0172 (14)	−0.0016 (12)
C5	0.0306 (15)	0.0631 (18)	0.0538 (17)	0.0011 (14)	−0.0059 (13)	0.0068 (14)
C6	0.0382 (16)	0.0432 (15)	0.0421 (15)	0.0115 (13)	0.0007 (13)	0.0056 (11)
C7	0.0513 (17)	0.0289 (13)	0.0404 (15)	0.0050 (12)	−0.0003 (12)	−0.0003 (10)
C8	0.0431 (15)	0.0360 (13)	0.0338 (13)	−0.0012 (12)	0.0014 (12)	0.0090 (10)
C9	0.056 (2)	0.0561 (17)	0.0530 (18)	0.0177 (16)	0.0161 (14)	0.0021 (14)
C10	0.0341 (14)	0.0767 (19)	0.0365 (14)	0.0078 (15)	0.0061 (12)	0.0012 (14)
C11	0.0330 (15)	0.0466 (15)	0.0281 (13)	−0.0016 (12)	−0.0026 (11)	0.0087 (11)
C12	0.0351 (15)	0.0496 (16)	0.0429 (16)	−0.0107 (13)	−0.0067 (12)	0.0127 (12)
C13	0.0544 (19)	0.0347 (14)	0.0404 (15)	−0.0060 (13)	−0.0051 (13)	−0.0017 (11)
C14	0.0388 (16)	0.0315 (12)	0.0372 (14)	0.0068 (12)	−0.0027 (11)	0.0004 (11)
C15	0.0270 (14)	0.0386 (13)	0.0353 (13)	0.0041 (11)	−0.0025 (11)	0.0011 (11)
C16	0.0300 (14)	0.0328 (12)	0.0283 (13)	0.0000 (11)	−0.0044 (11)	0.0015 (10)
C17	0.064 (2)	0.0572 (17)	0.0435 (17)	−0.0100 (16)	−0.0063 (14)	0.0125 (13)
N1	0.0609 (17)	0.0645 (16)	0.0575 (15)	−0.0188 (14)	0.0172 (13)	−0.0116 (12)
O1	0.0347 (11)	0.0791 (13)	0.0669 (14)	0.0165 (11)	−0.0073 (10)	−0.0073 (10)
O2	0.0593 (13)	0.0522 (11)	0.0543 (12)	−0.0180 (10)	−0.0204 (9)	−0.0005 (9)
O3	0.0377 (10)	0.0345 (8)	0.0344 (9)	0.0015 (8)	−0.0078 (8)	−0.0002 (7)
S1	0.0327 (3)	0.0438 (3)	0.0377 (3)	−0.0030 (3)	−0.0080 (3)	0.0024 (3)

### Geometric parameters (Å, º)

**Table:**

C1—C14	1.508 (4)	C10—H10A	0.9700
C1—C2	1.585 (4)	C10—H10B	0.9700
C1—H1C	0.9700	C11—C16	1.394 (3)
C1—H1D	0.9700	C11—C12	1.401 (4)
C2—C3	1.516 (4)	C12—C13	1.377 (4)
C2—H2A	0.9700	C12—H12	0.9300
C2—H2B	0.9700	C13—C14	1.384 (4)
C3—C4	1.387 (4)	C13—H13	0.9300
C3—C8	1.400 (3)	C14—C15	1.391 (3)
C4—C5	1.385 (4)	C15—C16	1.373 (3)
C4—H4	0.9300	C15—H15	0.9300
C5—C6	1.390 (4)	C16—O3	1.430 (3)
C5—H5	0.9300	C17—S1	1.742 (3)
C6—C7	1.389 (4)	C17—H17A	0.9600
C6—C9	1.509 (4)	C17—H17B	0.9600
C7—C8	1.394 (4)	C17—H17C	0.9600
C7—H7	0.9300	N1—H1A	0.8600
C8—N1	1.377 (3)	N1—H1B	0.8600
C9—C10	1.579 (4)	O1—S1	1.421 (2)
C9—H9A	0.9700	O2—S1	1.422 (2)
C9—H9B	0.9700	O3—S1	1.5908 (18)
C10—C11	1.499 (4)		
			
C14—C1—C2	111.5 (2)	C9—C10—H10A	109.0
C14—C1—H1C	109.3	C11—C10—H10B	109.0
C2—C1—H1C	109.3	C9—C10—H10B	109.0
C14—C1—H1D	109.3	H10A—C10—H10B	107.8
C2—C1—H1D	109.3	C16—C11—C12	114.1 (2)
H1C—C1—H1D	108.0	C16—C11—C10	123.2 (2)
C3—C2—C1	111.9 (2)	C12—C11—C10	120.9 (2)
C3—C2—H2A	109.2	C13—C12—C11	121.9 (2)
C1—C2—H2A	109.2	C13—C12—H12	119.1
C3—C2—H2B	109.2	C11—C12—H12	119.1
C1—C2—H2B	109.2	C12—C13—C14	121.0 (2)
H2A—C2—H2B	107.9	C12—C13—H13	119.5
C4—C3—C8	116.7 (2)	C14—C13—H13	119.5
C4—C3—C2	120.4 (2)	C13—C14—C15	116.7 (2)
C8—C3—C2	121.3 (2)	C13—C14—C1	121.3 (2)
C5—C4—C3	122.7 (3)	C15—C14—C1	120.9 (2)
C5—C4—H4	118.7	C16—C15—C14	120.1 (2)
C3—C4—H4	118.7	C16—C15—H15	119.9
C4—C5—C6	119.2 (3)	C14—C15—H15	120.0
C4—C5—H5	120.4	C15—C16—C11	122.9 (2)
C6—C5—H5	120.4	C15—C16—O3	118.5 (2)
C5—C6—C7	117.4 (2)	C11—C16—O3	117.7 (2)
C5—C6—C9	122.0 (3)	S1—C17—H17A	109.5
C7—C6—C9	119.2 (3)	S1—C17—H17B	109.5
C6—C7—C8	122.0 (3)	H17A—C17—H17B	109.5
C6—C7—H7	119.0	S1—C17—H17C	109.5
C8—C7—H7	119.0	H17A—C17—H17C	109.5
N1—C8—C7	119.3 (2)	H17B—C17—H17C	109.5
N1—C8—C3	121.1 (2)	C8—N1—H1A	120.0
C7—C8—C3	119.1 (3)	C8—N1—H1B	120.0
C6—C9—C10	113.6 (2)	H1A—N1—H1B	120.0
C6—C9—H9A	108.8	C16—O3—S1	117.52 (14)
C10—C9—H9A	108.8	O1—S1—O2	119.55 (14)
C6—C9—H9B	108.8	O1—S1—O3	109.55 (12)
C10—C9—H9B	108.8	O2—S1—O3	103.40 (10)
H9A—C9—H9B	107.7	O1—S1—C17	108.54 (14)
C11—C10—C9	113.1 (2)	O2—S1—C17	110.74 (14)
C11—C10—H10A	109.0	O3—S1—C17	103.86 (13)

### Hydrogen-bond geometry (Å, º)

**Table:**

*D*—H···*A*	*D*—H	H···*A*	*D*···*A*	*D*—H···*A*
N1—H1*B*···O2^i^	0.86	2.43	3.262 (3)	163
C10—H10*B*···O1^ii^	0.97	2.52	3.390 (4)	149

Symmetry codes: (i) −*x*+5/2, −*y*+1, *z*−1/2; (ii) *x*−1, *y*, *z*.
